# A comparison of pharmacoepidemiological study designs in medication use and traffic safety research

**DOI:** 10.1007/s10654-012-9689-3

**Published:** 2012-05-11

**Authors:** Silvia Ravera, Nienke van Rein, Johan J. de Gier, Lolkje T. W. de Jong-van den Berg

**Affiliations:** 1 Department of Pharmacotherapy and Pharmaceutical Care, University of Groningen, Antonius Deusinglaan 1, 9713 AV Groningen, The Netherlands; 2 Department of Pharmacoepidemiology and Pharmacoeconomics, University of Groningen, Antonius Deusinglaan 1, 9713 AV Groningen, The Netherlands

**Keywords:** Psychotropic medications, Road traffic accidents, Case–crossover study, Case–time–control study, Case–control study, Pharmacoepidemiology

## Abstract

In order to explore how the choice of different study designs could influence the risk estimates, a case–crossover and case–time–control study were carried out and their outcomes were compared with those of a traditional case–control study design that evaluated the association between the exposure to psychotropic medications and the risk of having a motor vehicle accident (MVA). A record-linkage database availing data for 3,786 cases and 18,089 controls during the period 2000–2007 was used. The study designs (i.e., case–crossover and case–time–control) were derived from published literature, and the following psychotropic medicines were examined: antipsychotics, anxiolytics, hypnotics and sedatives, and antidepressants, stratified in the two groups selective serotonin reuptake inhibitors (SSRIs) and other antidepressants. Moreover, in order to further investigate the effects of frequency of psychoactive medication exposure on the outcomes of the case–crossover analysis, the data were also stratified by the number of defined daily doses (DDDs) and days of medication use in the 12 months before the motor vehicle accident. Three-thousand seven-hundred fifty-two cases were included in this second part of the case–crossover analysis. The case–crossover design did not show any statistically significant association between psychotropic medication exposure and MVA risk [e.g., SSRIs—Adj. OR = 1.00 (95 % CI: 0.69–1.46); Anxiolytics—Adj. OR = 0.95 (95 % CI: 0.68–1.31)]. The case–time–control design only showed a borderline statistically significant increased traffic accident risk in SSRI users [Adj. OR = 1.16 (95 % CI: 1.01–1.34)]. With respect to the stratifications by the number of DDDs and days of medication use, the analyses showed no increased traffic accident risk associated with the exposure to the selected medication groups [e.g., SSRIs, <20 DDDs—Adj. OR = 0.65 (95 % CI: 0.11–3.87); SSRIs, 16–150 days—Adj. OR = 0.55 (95 % CI: 0.24–1.24)]. In contrast to the above-mentioned results, our recent case–control study found a statistically significant association between traffic accident risk and exposure to anxiolytics [Adj. OR = 1.54 (95 % CI: 1.11–2.15)], and SSRIs [Adj. OR = 2.03 (95 % CI: 1.31–3.14)]. Case–crossover and case–time–control analyses produced different results than those of our recent case–control study (i.e., case–crossover and case–time–control analyses did not show any statistically significant association whereas the case–control analysis showed an increased traffic accident risk in anxiolytic and SSRI users). These divergent results can probably be explained by the differences in the study designs. Given that the case–crossover design is only appropriate for short-term exposures and the case–time–control design is an elaboration of this latter, it can be concluded that, probably, these two approaches are not the most suitable ones to investigate the relation between MVA risk and psychotropic medications, which, on the contrary, are often use chronically.

## Introduction and aim

Driving a motor vehicle is a complex task that involves several psychomotor and cognitive skills [1]. Some commonly prescribed medications can influence cognitive and psychomotor functions and, therefore, impair the ability to drive safely [1, 2].

The risk of experiencing a road traffic accident while exposed to psychotropic medications has often been estimated by means of pharmacoepidemiological studies, and, in particular, mainly by case–control and case–crossover studies [3]. The results of these studies have frequently shown a positive association between the risk of having a motor vehicle accident (MVA) and the exposure to some groups of psychoactive medications (e.g., benzodiazepines, benzodiazepine-like substances such as zopiclone and zolpidem, tricyclic antidepressants) [3–5], but, in some cases, their findings have been rather controversial. For instance, in 1997, Hemmelgarn et al. [6] performed a case–control study which showed that elderly drivers exposed to long half-life benzodiazepines (BZDs) were significantly associated to the risk of having an MVA within the first week of benzodiazepine use, but, on the contrary, in 1998, the case–crossover study of Barbone et al. [7] found no increased traffic accident risk associated to benzodiazepine use in individuals ≥65 years old. A similar discrepancy was also described in the study of Hebert et al. [8] which showed an increased MVA in case of long half-life BZD elderly users by applying a case–control approach, but no association was found by using a case–crossover analysis. Another example is a recent Dutch case–control study [9] which reported a statistically significant association between the risk of experiencing a traffic accident and the exposure to selective serotonin reuptake inhibitors (SSRIs); however, these results differed from those of Barbone’s case–crossover study, which found no increased MVA risk in SSRI users [7].

The divergences in the outcomes of these pharmacoepidemiological studies could be explained by the use of different study designs. Generally speaking, case–control studies compare cases with an event to controls without the event, looking for differences in the antecedent exposures [10]. Case–control studies can be useful when assessing a wide range of possible causes of a single event as well as the evaluation of relatively rare events [10, 11]. However, one of the limitations that are often encountered while using this study design is the selection of the controls, which can lead to selection bias and, consequently, incorrect conclusions [10, 11]. One possible alternative to the case–control design is the case–crossover design. The case–crossover design is an adaptation of the case–control design in which cases serve as their own controls [12–14]. Because of this peculiarity, the case–crossover design is immune to the control-selection bias, which, as stated above, could hamper case–control studies, and it also controls for stable subject-specific covariates [12, 14, 15]. However, the case–crossover design is only appropriate to investigate the effects of incidental exposures on the event of interest and, therefore, is not suitable to estimate the risk in people exposed to long-term treatments [7, 15, 16]. If properly designed and performed, both study designs are valuable research tools; nevertheless, due to their assumptions, strengths and limitations, caution has to be applied when interpreting and comparing their results [11].

Given the fact that the exposure to medications may change over time [17], it seems reasonable to take the case–time–control design into consideration, as well. This type of epidemiologic study design can be regarded as an extension of the case–crossover design which uses, in addition to the case group, a series of controls to adjust for exposures that vary over time [18, 19], and, therefore, it can offer a useful approach to eliminate the biasing effect of the aforementioned confounding factor [20].

The aim of this study was to assess the effects of different study designs on the risk estimate. To do so, a case–crossover and case–time–control study were carried out and their outcomes were compared with those of a traditional case–control study design that evaluated the association between the risk of having a motor vehicle accident and the exposure to some psychotropic medication groups (which are known to be related to driving impairment [3, 5, 7, 21]) [9].

## Methods

The case–crossover study, linking police traffic accident and pharmacy prescription databases, was performed in the Netherlands, and was focused on a 7-year period (1st January 2000–31st December 2007).

The data sources, inclusion and exclusion criteria, and exposure definition have been described in detail elsewhere [9]. In brief, a Trusted Third Party (TTP) performed the linkage between the PHARMO [24], Dutch Traffic and Navigation Authority (DVS) [25], and Dutch Road Transport Authority (RDW) [26] databases, which provided pharmacy prescription data (in particular, the following details were available: dispensing date, the prescribed dosage, the dispensed quantity and the estimated duration of use), traffic accident data, and driving license records, respectively. Cases were defined as drivers who had an MVA attended by the Dutch police during the study time–frame. Subjects were excluded if they were ≤18 years old at the time of the accident (i.e., index date) and if they tested positive for alcohol or no alcohol test data were available.

The following medication groups were evaluated: antipsychotics (ATC code: N05A), anxiolytics (ATC code: N05B), hypnotics and sedatives (ATC code: N05C), antidepressants stratified in selective serotonin reuptake inhibitors (SSRIs) (ATC code: N06AB), and other antidepressants [i.e. non-selective monoamine reuptake inhibitors (ATC code: N06AA), monoamine oxidase A inhibitors (MAOs) (ATC code: N06AG), other antidepressants (ATC code: N06AX)].

The case window was defined as the week before the index date whereas the control window was defined as the same week 1 year before the index date, to control for possible seasonal and weather variations which could play a causal role in traffic accidents.

Exposure was considered to start the day after the dispensing date. Medications dispensed on the MVA day were not included because it was not possible to determine whether, in the case window, exposure occurred before or after the traffic accident. Subjects were considered to be exposed if the medication was used during the week before the index date; if the medication exposure ended 2 days before the index date, the subjects were still considered as exposed.

**Fig. 1:**
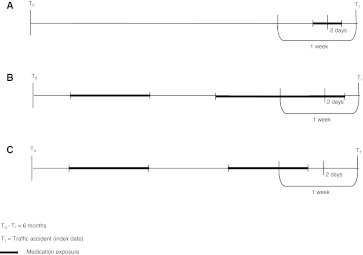
Medication exposure in the case window (a 6-month period was considered). *A* Acute user—Exposed; *B* Regular user—Exposed; *C* Regular user—Not exposed. It is important to note that the same procedure was followed to assess medication exposure in the control window (i.e., 1 year before the index date)

In order to evaluate the effects of the user type on the results of the case–crossover design, the study population was stratified as follows: (1) Regular users: subjects who were exposed to a driving impairing medication in the week before the index date and also used the same medication in the 6 months before the index date (i.e., subjects who used a psychotropic medicine on a regular basis during the 6 months preceding the traffic accident); (2) Acute users: subjects who used a driving impairing medication in the week before the index date, but did not receive any prescriptions for the same medication in the 6 months before the initiation of the therapy (i.e., subjects who initiated their therapy in the week before the MVA, but did not use this medication in the 6 months before the initiation of the therapy). In this analysis, subjects were excluded if their medication history in the 18 months preceding the index date was not available (Fig. 1).

In order to account for the potential time trends in psychotropic medication use in the case and control window [22, 23], a case–time–control analysis was also performed using the same control group that was used in the case–control study mentioned above [9]. For this investigation, a control group of 18,089 subjects was used. In brief, the selected controls had to be ≥19 years old, be in possession of a driving license and have had no traffic accident during the study period. Four controls were matched for each case; the matching was by gender, age within 5 years, zip code, and date of the accident of the correspondent case. The definitions of the case and control windows and exposure were the same as reported above.

In order to further investigate the effects of frequency of psychoactive medication exposure on the outcomes of the case–crossover analysis, subjects were also stratified by the number of defined daily doses (DDDs) (i.e., the total number of assumed average maintenance doses per day that the subjects used in the 12 months preceding the index date, up to and including the week before the MVA) and days of medication use in the 12 months before the index date (i.e., the total number of days of therapy during the 12 months preceding the index date, up to and including the week before the MVA), with the purpose of having a broader overview of the subjects’ medication exposures preceding their traffic accidents. As a consequence, in this analysis, cases were excluded if their medication history in the 2 years preceding the index date was not available.

Descriptive statistics was used to describe the demographic characteristics of the cases and controls as well as the accident characteristics of the cases.

For the case–crossover and case–time–control designs, logistic regression analysis was used to estimate odds ratios (ORs) and 95 % confidence intervals (95 % CIs). The standard method for matched case–control studies was used in order to calculate the ORs. The ORs were the measure of the odds of exposure in the case window versus the control window; specifically, medication exposure in the week before the MVA (case window) was compared with medication exposure during the same week of the control window, 1 year earlier.

Adjusted ORs were calculated by including exposure to combination therapy (i.e., concomitant use of at least two medicines) in the model.

A “control–crossover” analysis was performed similarly for the selected control group.

The case–time–control ORs were estimated by dividing the case–crossover ORs from the cases by “control–crossover” ORs from the controls.

All statistical analyses were performed by using the statistical package PASW Statistics Version 18.

The study research protocol was reviewed by the Medical Ethics Committee of the University Medical Centre Groningen (UMCG), the Netherlands, which resulted in the decision that, according the Dutch Medical Research Involving Human Subjects Act (WOM), this study did not need an ethical approval.

## Results

Three-thousand seven-hundred eighty-six cases were included in the first part of the case–crossover analysis.

The demographic characteristics of the cases included the case–crossover study are presented in Table 1. As shown in this table, the majority of case population was male (62.3 %) and the age group 30–60 was the most represented one (54.2 %).

**Table 1:** Demographic characteristics of motor vehicle accident of the cases

Cases characteristics (n = 3,786)	N (%)
*Gender*	
Male	2,360 (62.3)
Female	1,426 (37.7)
*Age (years)*	
<30	1,062 (28.1)
30–60	2,051 (54.2)
≥61	673 (17.8)

Table 2 illustrates the characteristics of the case accidents (i.e., season, weather conditions, time of the week and time of the day, light conditions, and severity of the MVA). Accidents were almost equally distributed during the four seasons, mainly occurred during week days, with dry weather conditions, at daylight, between 1 p.m. and 7 p.m., and were mostly either severe or moderately severe.

**Table 2:** Characteristics of the accidents of motor vehicle accident of the cases

Accident characteristics (N = 3,786)	N (%)
*Season*	
Winter	916 (24.2)
Spring	969 (25.6)
Summer	850 (22.4)
Autumn	1,051 (27.8)
*Weather*	
Dry	3,067 (81.0)
Rain	599 (15.8)
Snow/Hail	45 (1.2)
Fog	49 (1.3)
Hard wind	2 (0.1)
Unknown	24 (0.6)
*Week/Weekend*	
Week day	2,911 (76.9)
*Time*	
1 a.m.–7 a.m.	239 (6.3)
7 a.m.–1 p.m.	1,203 (31.8)
1 p.m.–7 p.m.	1,714 (45.3)
7 p.m.–1 a.m.	630 (16.6)
*Light*	
Daylight	2,741 (72.4)
Dark	826 (21.8)
Dawn	219 (5.8)
*Severity*	
Fatal	24 (0.6)
Severely injured (Hospitalization >24 h)	1,321 (34.9)
Moderately injured (1st aid point or hospitalization <24 h)	1,421 (37.5)
Slightly injured (Treated on scene)	1,020 (26.9)

Table 3 presents the medication exposure of the cases (regular users and acute users) and controls (regular users and acute users), in the case and control windows, and the case–crossover and case–time–control crude and adjusted ORs for road-traffic accidents related to the exposure to the selected psychoactive medication groups.

**Table 3:** Number and percentage of cases and controls exposed to different psychotropic medication groups, in the case and control windows, and case–crossover and case–time–control crude and adjusted ORs (with 95 % confidence intervals) for road-traffic accidents in different psychotropic medicine group users (ATC codes in brackets), stratified by regular users and acute users

Medicine group	Exposed in case window (%)	Exposed in control window (%)	Case–crossover crude OR (95 % CI)	Case–crossover Adj. OR (95 % CI)	Case–time–control crude OR (95 % CI)	Case–time–control Adj. OR (95 % CI)
*Antipsychotics (N05A)*						
Cases (N = 3,786)						
Regular users	18 (0.50)	23 (0.60)	0.76 (0.41–1.41)	0.68 (0.34–1.35)	0.94 (0.67–1.32)	0.86 (0.61–1.23)
Acute users	1 (0.02)	1 (0.02)	0.97 (0.06–15.52)	0.97 (0.06–15.52)	1.01 (0.43–2.27)	0.50 (0.33–0.73)
Controls (N = 3,786)						
Regular users	91 (0.50)	108 (0.60)	0.81 (0.61–1.07)	0.79 (0.56–1.10)	–	–
Acute users	2 (0.01)	2 (0.01)	0.96 (0.14–6.84)	1.93 (0.18–21.26)	–	–
*Anxiolitics (N05B)*						
Cases						
Regular users	92 (2.40)	94 (2.50)	0.95 (0.71–1.27)	0.95 (0.68–1.31)	1.09 (0.95–1.25)	1.10 (0.94–1.27)
Acute users	13 (0.34)	11 (0.29)	1.15 (0.51–2.56)	0.97 (0.40–2.33)	1.28 (0.88–1.86)	1.04 (0.70–1.52)
Controls						
Regular users	303 (1.70)	335 (1.90)	0.87 (0.75–1.02)	0.86 (0.72–1.03)	–	–
Acute users	40 (0.22)	43 (0.24)	0.90 (0.58–1.38)	0.93 (0.57–1.53)	–	–
*Hypnotics (N05C)*						
Cases						
Regular users	75 (2.00)	85 (2.20)	0.86 (0.63–1.17)	0.89 (0.63–1.25)	0.98 (0.84–1.13)	0.95 (0.81–1.12)
Acute users	6 (0.16)	11 (0.29)	0.53 (0.20–1.43)	0.39 (0.12–1.24)	0.88 (0.59–1.36)	0.49 (0.28–0.85)
Controls						
Regular users	268 (1.50)	293 (1.60)	0.88 (0.75–1.04)	0.94 (0.78–1.12)	–	–
Acute users	20 (0.11)	32 (0.18)	0.60 (0.34–1.05)	0.80 (0.43–1.46)	–	–
*SSRIs (N06AB)*						
Cases						
Regular users	92 (2.40)	87 (2.30)	1.03 (0.76–1.38)	1.00 (0.69–1.46)	**1.26 (1.10–1.41)**	**1.16 (1.01–1.34)**
Acute users	7 (0.18)	5 (0.13)	1.36 (0.43–4.28)	1.29 (0.29–5.79)	1.19 (0.84–1.69)	1.07 (0.60–1.90)
Controls						
Regular users	240 (1.30)	281 (1.60)	0.82 (0.69–0.98)	0.86 (0.68–1.09)	–	–
Acute users	13 (0.07)	11 (0.06)	1.14 (0.51–2.54)	1.21 (0.48–3.05)	–	–
*Other antidepressants*						
Cases						
Regular users	40 (1.10)	45 (1.20)	0.86 (0.56–1.33)	0.88 (0.53–1.46)	1.10 (0.90–1.37)	1.24 (0.98–1.55)
Acute users	3 (0.08)	3 (0.08)	0.97 (0.20–4.81)	0.97 (0.20–4.81)	**2.37 (1.25–4.45)**	**1.76 (1.11–3.01)**
Controls						
Regular users	143 (0.80)	177 (1.00)	0.78 (0.62–0.97)	0.71 (0.54–0.94)	–	–
Acute users	6 (0.03)	14 (0.08)	0.41 (0.16–1.08)	0.55 (0.18–1.60)	–	–

*Crude OR* Crude odds ratio and corresponding 95 % confidence interval (95 % CI)

*Adj. OR* Adjusted odds ratio and corresponding 95 % confidence interval (95 % CI) (ORs were adjusted for combination therapy—i.e., the concomitant use of at least two study medicines)

*SSRIs* Selective serotonin reuptake inhibitors

Bold: Statistically significant

From this table it can be seen that, in the case group, anxiolytics and SSRIs were the two most used medication classes, with the exception of the control window of acute users (in this case, hypnotics and anxiolytics were the most represented classes). On the contrary, in the control group, the two most represented medication classes were anxiolytics and hypnotics, with the exception of the control window of regular users (in this case, anxiolytics were the most represented classes, followed by hypnotics and SSRIs which reported the same percentage of users).

With respect to the crude and adjusted ORs for road-traffic accidents related to the exposure to the selected psychoactive medication groups, it can be seen that the case–crossover analysis did not show any statistically significant association between MVA risk and the exposure to the selected medications.

After dividing the ORs in the cases by the ORs in the controls (case–time–control analysis), a significant increased traffic accident risk was obtained for the SSRIs, if regular users were taken into consideration, whereas a statistically significant association was found between other antidepressants and MVA risk, if the analysis was restricted to acute users (see Table 3, last right-hand column).

Three-thousand seven-hundred fifty-two cases were included in the second part of the case–crossover analysis (see Table 4—crude and adjusted ORs for road-traffic accidents in different medication group users, stratified by the number of DDDs and days of medication use in the year before the traffic accident). As can be seen from Table 4, our analyses showed no increased traffic accident risk associated with the exposure to the selected medication groups stratified by days of use and DDDs in the year preceding the index date.

**Table 4:** Number of motor vehicle accident cases exposed to different psychotropic medication groups (ATC codes in brackets), in the case and control windows, stratified by number of days of use and number of DDDs, and case–crossover crude and adjusted ORs (with 95 % confidence intervals) for road-traffic accidents in the year before the index date (N = 3,752)

Medicine group	Exposed in case window	Exposed in control window	Case–crossover crude OR (95 % CI)	Case–crossover Adj. OR (95 % CI)
*Antipsychotics (N05A)*				
1–15 days	0	1	–	–
16–150 days	1	3	0.32 (0.03–3.11)	0.32 (0.03–3.11)
≥151 days	17	19	0.87 (0.45–1.67)	0.79 (0.38–1.64)
<20 DDDs	1	2	0.48 (0.04–5.35)	0.48 (0.04–5.35)
21–150 DDDs	6	9	0.66 (0.23–1.82)	0.48 (0.15–1.61)
≥151 DDDs	11	12	0.89 (0.39–2.02)	0.87 (0.35–2.15)
*Anxiolytics (N05B)*				
1–15 days	11	8	1.33 (0.54–3.32)	1.45 (0.52–4.09)
16–150 days	26	37	0.68 (0.41–1.13)	0.59 (0.34–1.05)
≥151 days	54	49	1.07 (0.72–1.58)	1.12 (0.73–1.72)
<20 DDDs	22	27	0.79 (0.45–1.39)	0.78 (0.41–1.49)
21–150 DDDs	40	40	0.97 (0.62–1.51)	0.94 (0.59–1.51)
≥151 DDDs	29	27	1.04 (0.61–1.76)	1.07 (0.58–1.96)
*Hypnotics (N05C)*				
1–15 days	5	7	0.69 (0.22–2.18)	0.65 (0.18–2.29)
16–150 days	15	28	0.52 (0.28–0.97)	0.57 (0.29–1.14)
≥151 days	55	50	1.07 (0.72–1.57)	1.08 (0.71–1.64)
<20 DDDs	6	10	0.58 (0.21–1.60)	0.61 (0.20–1.85)
21–150 DDDs	15	28	0.52 (0.28–0.97)	0.53 (0.27–1.03)
≥151 DDDs	54	47	1.11 (0.75–1.65)	1.16 (0.76–1.79)
*SSRIs (N06AB)*				
1–15 days	4	4	0.97 (0.24–3.88)	0.65 (0.11–3.87)
16–150 days	13	25	0.50 (0.26–0.99)	0.55 (0.24–1.24)
≥151 days	75	58	1.25 (0.89–1.77)	1.23 (0.80–1.92)
<20 DDDs	4	4	0.97 (0.24–3.88)	0.65 (0.11–3.87)
21–150 DDDs	13	24	0.53 (0.27–1.03)	0.58 (0.25–1.33)
≥151 DDDs	75	59	1.23 (0.87–1.74)	1.20 (0.78–1.86)
*Other antidepressants*				
1–15 days	1	2	0.48 (0.04–5.35)	0.97 (0.06–15.50)
16–150 days	7	7	0.97 (0.34–2.77)	0.65 (0.18–2.29)
≥151 days	31	34	0.88 (0.54–1.44)	0.93 (0.52–1.65)
<20 DDDs	2	4	0.48 (0.09–2.65)	0.65 (0.11–3.87)
21–150 DDDs	20	19	1.02 (0.53–1.92)	1.04 (0.50–2.15)
≥151 DDDs	17	20	0.82 (0.43–1.58)	0.76 (0.35–1.68)

*Crude OR* Crude odds ratio and corresponding 95 % confidence interval (95 % CI), *Adj. OR* Adjusted odds ratio and corresponding 95 % confidence interval (95 % CI) (ORs were adjusted for combination therapy—i.e., the concomitant use of at least two study medicines), *SSRIs* Selective serotonin reuptake inhibitors, *DDDs* Defined daily doses

In contrast to the above-mentioned results, our recent case–control study found a statistically significant association between traffic accident risk and exposure to anxiolytics [Adj. OR = 1.54 (95 % CI: 1.11–2.15)], and SSRIs [Adj. OR = 2.03 (95 % CI: 1.31–3.14)] [9].

## Discussion and conclusions

To the best of our knowledge, this is one of the few studies that evaluated and highlighted the possible impact of different epidemiologic study designs (i.e., case–control, case–crossover, and case–time–control) on the association between MVA risks and psychotropic medication exposure in the same study population.

The results of our case–crossover study did not show any significant increase in MVA risk associated with the exposure to the selected psychotropic medicine groups [e.g., Regular user stratification: Anxiolytics: Adj. OR = 0.95 (95 % CI: 0.68–1.31); SSRIs: Adj. OR = 1.00 (95 % CI: 0.69–1.46)]. Stratifications according to the number of days and DDDs used in the previous year were consistent with the above-mentioned findings, and, in particular, did not show any effects of exposure frequency on the risk of experiencing an MVA [e.g., 1–15 day stratification: Anxiolytics: Adj. OR = 1.45 (95 % CI: 0.52–4.09); SSRIs: Adj. OR = 0.65 (95 % CI: 0.11–3.87)]. Therefore, if compared to our recent pharmacoepidemiological study [9], it can be observed that the current case–crossover analysis produced different results than those of the case–control analysis, which actually found a statistically significant association between traffic accident risk and exposure to anxiolytics and SSRIs [Anxiolytics: Adj. OR = 1.54 (95 % CI: 1.11–2.15); SSRIs: Adj. OR = 2.03 (95 % CI: 1.31–3.14)—all exposed individuals].

Lastly, the outcomes of the case–time–control analysis showed a borderline statistically significant increased risk only in SSRI users, in the stratification referred to regular users [Adj. OR = 1.16 (95 % CI: 1.01–1.34)], whereas the acute user stratification only showed a statistically significant association between MVA risk and other antidepressant users [Adj. OR = 1.76 (95 % CI: 1.11–3.01)]. Therefore, it can be speculated that, in this case, the findings of the case–time–control analysis only partially supported the outcomes of the case–control one.

The discrepancies between the outcomes of the case–control and case–crossover studies could be attributed to the choice of study design. The case–crossover design is a commonly used scientific method to investigate whether a certain event was triggered by something unusual that happened just before the event itself [14]. The case–crossover is a matched case–control study, but it only involves cases and each case serves as its own control [14]. Because of this peculiarity, the case–crossover design controls for stable subject-specific covariates and it overcomes control selection bias [13]. However, this type of design requires that the exposures are brief and their effects transient [10, 13]. Considering that psychotropic medications are often used on a regular and chronic basis [8, 17, 27], it can be speculated that, in the present study, one of the most important assumptions of the case–crossover design was not met, and, therefore, the choice of this study design was probably not appropriate. To be more precise, it is relevant to point out that the case–crossover odds ratio is estimated by the ratio of the number of cases exposed only during the case window to the number of cases exposed only during the control window (i.e., ratio of discordant pairs). Given that only discordant pairs contribute to the estimation of the odds ratio in matched analyses, if the exposure does not change in a systematic way over time, it is likely to face a loss of precision because there is a lack of discordant pairs as exposure becomes more homogeneous, and eventually reduces the power of the study [13, 22, 29, 30]. Therefore, based on the above-mentioned considerations, it can be conceivably hypothesised that the case–crossover analysis should be limited to intermittent users of the selected medication groups. However, it is important to note that, in the current study, this restriction led to a consistent loss of cases and, even if the ORs calculated for this specific group of users were more similar to the ORs obtained by applying the case–control technique, it can be speculated that, as stated before, our study did not have adequate statistical power to detect reliably the association between incidental psychotropic medication users and MVA risks [10, 11].

Stratifying the data according to the number of DDDs and days of use in the previous year did not support the associations that were shown in the case–control study either. With respect to the DDD, a possible explanation for this might be that, since the defined daily dose is a unit of measurement and does not necessarily reflect the recommended or prescribed daily dose [28], the actual doses used by our study population could have been considerably different from the recommended DDD; therefore, perhaps this stratification was not appropriate and led to a misclassification of our medication users.

With respect to the days of use, it is difficult to explain the study outcomes, but, as stated above, they could be related to the low sample size in the infrequent user groups which might have resulted in a lack of statistical power to address the issue of the association between the risk of experiencing an MVA while incidentally exposed to psychoactive medications [10, 11].

Besides the points reported above, there could also be other possible explanations for the discrepancies among the findings of the two designs that were used. As some authors have also pointed out [8, 13, 22, 29, 30], possible reasons for different results between case–crossover and case–control studies may be related to selection bias of the control–person–time (i.e., our selected control–person–time did not properly represent the population-time that generated the cases due to, for example, possible divergences in the driving patterns between the case and control times), confounding by indication (no information was available on what medical condition the psychotropic medications were prescribed for, and, consequently, we could not account for the confounding effect of the disease) different effects of the medication at different points in time (e.g., different estimates in relation to therapy duration and/or prior exposures [31]), time-varying within-subject confounding factors (e.g., fluctuations in disease severity, co-morbidities, etc.), and time trend bias (i.e., changes in the prescribing patterns of the medications of interest).

With regard to the case–time–control analysis, our study only showed a positive association between MVA risk and SSRI users [Adj. OR = 1.16 (95 % CI: 1.01–1.34)], in the regular user group, and other antidepressant users [Adj. OR = 1.76 (95 % CI: 1.11–3.01)], in the acute user stratification, but, in contrast to our earlier findings, no evidence of an increased traffic accident risk associated with anxiolytics was detected [Adj. OR = 1.10 (0.94–1.27)]. The reason for the discrepant outcomes of this analysis is not clear, but it might also be related to the choice of the study design. The current case–time–control study was performed to remove bias due to time trends from the case–crossover estimate [22, 23], and, as suggested by Suissa [18], to possibly control for confounding by indication. However, since the case–time–control design can be seen as an elaboration of the case–crossover design [30] (i.e., it corresponds to the division of the case–crossover matched-pair odds ratio by a “control–crossover” (time–control) matched-pair odds ratio [32]), our findings could have been limited by the same shortcomings as those of the case–crossover approach (e.g., selection bias in the control–time window, within-person confounding, time-varying within-subject confounding factors, etc.). Additionally, our case–time–control design might have had the same difficulty addressing chronic exposures and chronic effects as our case–crossover analysis. In particular, if the exposure was chronic, few controls were available with discordant exposures in different time periods, and, as well as the case–crossover design, our resultant case–time–control analysis could have been hampered by a poor statistical power compared to a conventional study [32]. Moreover, since the case–time–control design requires a traditional control group, our study, and, consequently, its results could have been hampered by the same limitations as the case–control design, as well (e.g., selection bias in the collection process of the control group, between-person confounding, higher complexity due to the necessity of a control group, etc.) [18, 22, 30]. Lastly, as Greenland argued [32], on the one hand, our case–time–control design could have been a helpful tool to adjust for time trends in measured exposures, but, on the other hand, if unmeasured confounders and/or carryover effects were present, new biases could have been introduced. As a consequence, the problem of confounding by indication would not have been solved and our final results could have been either more or less confounded than those obtained by the case–control and case–crossover analyses [32].

Our study supports the observations of Hebert et al. [8], who also compared the results of a case–control study to those of a case–crossover study using the same database to determine the association between BZDs and the risk of MVAs. In that study, the case–control approach demonstrated an increased MVA risk associated with the use of long-acting BZDs whereas the case–crossover approach applied to all cases did not show any association. The authors concluded that the differences among the findings of these studies could have derived from intrinsic differences between the two designs, and that, in particular, a lack of intermittency of exposure could have altered the point estimates of their case–crossover analysis [8].

Although the differences between the study populations should be considered as a possible cause of divergent findings, the previously mentioned assumption could also clarify the discrepancies between the outcomes of Hemmelgarn et al.’s case–control study [6] and those of Barbone et al.’s case–crossover study [7] which, respectively, showed a statistically significant association between BZD exposure and traffic accident in older adults and no evidence that BZDs increased traffic accident risks in elderly patients.

Lastly, this hypothesis could also explain the contradictory findings between our case–control study on SSRIs and increased MVA risk [9] and Barbone’s case–crossover outcomes which, in contrast to our research, found no increased risk of road-traffic accidents in users of SSRIs [7].

In conclusion, our investigation has shown that different study designs seemed to give different answers to the same research hypothesis, in the same population (i.e., the outcomes of the case–crossover and case–time–control analyses were not in line with the outcomes of the case–control analysis, which showed an increased traffic accident risk in anxiolytic and SSRI users). Considering that every study design has different design-specific assumptions, and strengths and limitations, it could be assumed that our analyses actually tested distinctive causal hypotheses and focused on different aspects of psychoactive medication use and MVA risk [8, 22, 29]. As a consequence, it seems reasonable to conclude that each pharmacoepidemiological design may be appropriate only in certain settings and under specific assumptions [22], and, therefore, if possible, multiple designs and analyses should be used to investigate the different aspects of factors that can play a role in traffic safety while driving under the influence of psychotropic medications.
